# 1,2-Bis(benz­yloxy)-1,2-bis­(4-chloro­phen­yl)-3,8-dimeth­oxy­acenaphthene

**DOI:** 10.1107/S1600536811035495

**Published:** 2011-09-03

**Authors:** Teruhisa Takada, Daichi Hijikata, Akiko Okamoto, Hideaki Oike, Noriyuki Yonezawa

**Affiliations:** a Department of Organic and Polymer Materials Chemistry, Tokyo University of Agriculture & Technology, Koganei, Tokyo 184-8588, Japan

## Abstract

In the title compound, C_40_H_32_Cl_2_O_4_, the two chloro­benzene rings are in *syn* orientations with respect to the naphthalene ring system and make dihedral angles of 57.12 (6) and 85.74 (6)° with it. The benzene rings of the benz­yloxy group make dihedral angles of 75.34 (6) and 83.95 (7)°, with the naphthalene ring system. In the crystal, the mol­ecules are linked by inter­molecular C—H⋯Cl inter­actions between the methyl­ene H atoms of the benz­yloxy group and the Cl atoms in adjacent mol­ecules. Furthermore, centrosymmetrically related mol­ecules are linked into dimeric units by pairs of C—H⋯π inter­actions.

## Related literature

For the synthesis of aroylated naphthalene compounds *via* electrophilic aromatic substitution of naphthalene derivatives, see: Okamoto & Yonezawa (2009 ▶). For the structures of closely related compounds, see: Watanabe *et al.* (2010*a* ▶,*b* ▶); Mitsui *et al.* (2010 ▶); Hijikata *et al.* (2010 ▶); Nakaema *et al.* (2007 ▶).
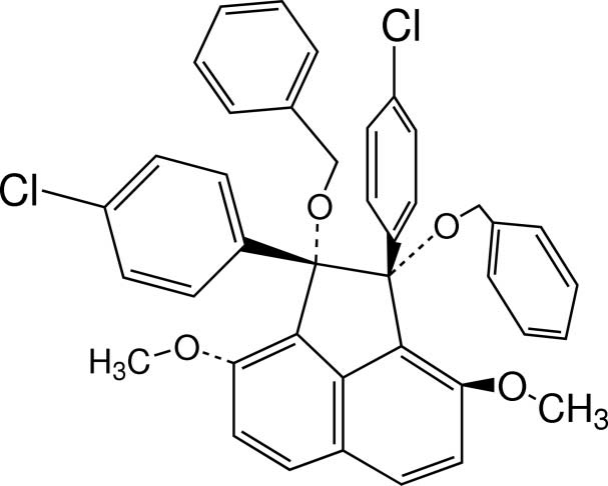


## Experimental

### 

#### Crystal data

C_40_H_32_Cl_2_O_4_
*M* *_r_* = 647.56
Triclinic, 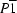

*a* = 10.9773 (2) Å
*b* = 12.6514 (2) Å
*c* = 12.9171 (2) Å
α = 102.387 (1)°
β = 104.899 (1)°
γ = 103.306 (1)°
*V* = 1614.04 (5) Å^3^
*Z* = 2
Cu *K*α radiation
μ = 2.15 mm^−1^
*T* = 193 K
0.50 × 0.30 × 0.20 mm

#### Data collection

Rigaku R-AXIS RAPID diffractometer
Absorption correction: numerical (*NUMABS*; Higashi,1999 ▶) *T*_min_ = 0.414, *T*_max_ = 0.674
30622 measured reflections
5828 independent reflections
5503 reflections with *I* > 2σ(*I*)
*R*_int_ = 0.061

#### Refinement

*R*[*F* ^2^ > 2σ(*F* ^2^)] = 0.037
*wR*(*F* ^2^) = 0.107
*S* = 1.06
5828 reflections
418 parameters
H-atom parameters constrained
Δρ_max_ = 0.43 e Å^−3^
Δρ_min_ = −0.31 e Å^−3^

### 

Data collection: *PROCESS-AUTO* (Rigaku, 1998 ▶); cell refinement: *PROCESS-AUTO*; data reduction: *CrystalStructure* (Rigaku/MSC, 2004 ▶); program(s) used to solve structure: *SIR2004* (Burla *et al.*, 2005 ▶); program(s) used to refine structure: *SHELXL97* (Sheldrick, 2008 ▶); molecular graphics: *ORTEPIII* (Burnett & Johnson, 1996 ▶); software used to prepare material for publication: *SHELXL97*.

## Supplementary Material

Crystal structure: contains datablock(s) I, global. DOI: 10.1107/S1600536811035495/jh2320sup1.cif

Structure factors: contains datablock(s) I. DOI: 10.1107/S1600536811035495/jh2320Isup2.hkl

Supplementary material file. DOI: 10.1107/S1600536811035495/jh2320Isup3.cml

Additional supplementary materials:  crystallographic information; 3D view; checkCIF report

## Figures and Tables

**Table 1:** Hydrogen-bond geometry (Å, °) *Cg*6 is the centroid of the C35–C40 ring.

*D*—H⋯*A*	*D*—H	H⋯*A*	*D*⋯*A*	*D*—H⋯*A*
C34—H34*A*⋯Cl1^i^	0.99	2.66	3.4748 (16)	140
C16—H16⋯*Cg*6^ii^	0.95	2.70	3.3962 (16)	131

Symmetry codes: (i) 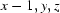; (ii) 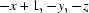.

**Fig. 1.**
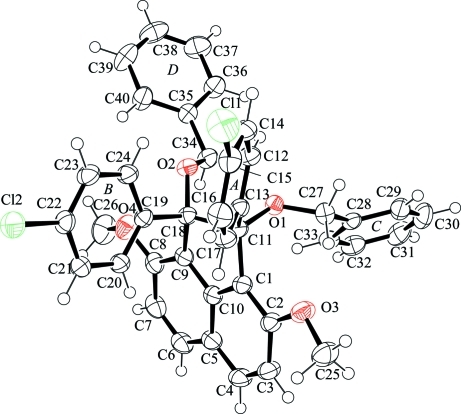
The molecular structure of the title molecule, showing 50% probability displacement ellipsoids.

**Fig. 2.**
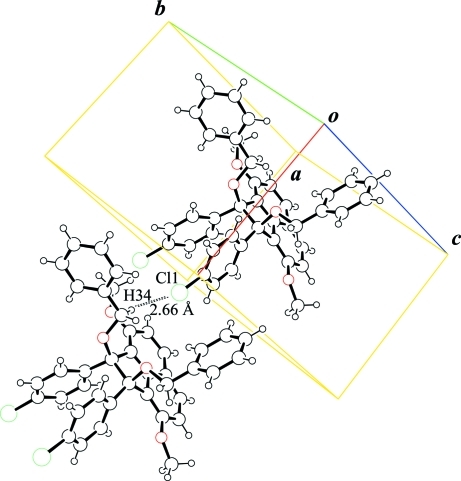
A dimeric pair of the title molecules, showing the intermolecular C—H···Cl interactions as double dashed line [symmetry code: (i) x - 1, y, z].

**Fig. 3.**
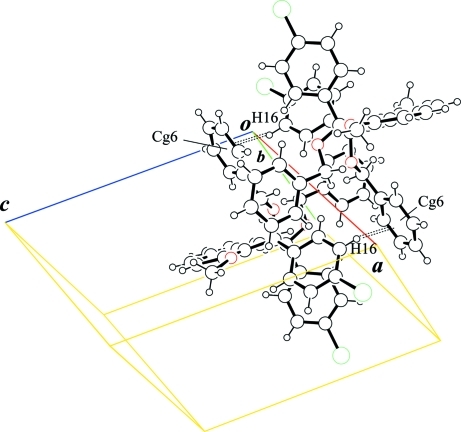
A dimeric pair of the title molecules. The intermolecular C—H···π interactions are observed along c axis (double dashed lines) [symmetry code: (ii) -x + 1, -y, -z].

## supplementary crystallographic information

### Comment

In the course of our study on electrophilic aromatic aroylation of 2,7-dimethoxynaphthalene, *peri-*aroylnaphthalene compounds have proven to be formed regioselectively with the aid of suitable acidic mediators (Okamoto & Yonezawa, 2009). Recently, we have reported the crystal structures of several 1,8-diaroylated naphthalene homologues exemplified by bis(4-fluorophenyl)(2,7-dimethoxynaphthalene-1,8-diyl)dimethanone (Watanabe *et al.*, 2010*a*), and bis(4-bromophenyl)(2,7-dimethoxynaphthalene-1,8-diyl)dimethanone (Watanabe *et al.*, 2010*b*). The aroyl groups at the 1,8-positions of the naphthalene rings in these compounds are twistedly bonded in an almost perpendicular fashion, but the benzene ring moieties of the aroyl groups tilt slightly toward the *exo* sides of the naphthalene rings. On the other hand, 1,8-bis(4-chlorobenzoyl)-7-methoxynaphthalene-2-ol ethanol monosolvate (Mitsui *et al.*, 2010) and 2,7-dimethoxy-1,8-bis(4-phenoxybenzoyl)naphthalene (Hijikata *et al.*, 2010) have been revealed that the aroyl groups attached to the naphthalene ring are oriented in the same direction, *i.e.*, *syn*-orientation. As a part of our continuous study on the molecular structures of this kind of homologous molecules, the X-ray crystal structure of the title compound, acenaphthene derivative bearing benzyloxy and 4-chlorophenyl groups, is discussed in this article. The title compound was prepared by Zn-complex-mediated pinacol coupling of 1,8-bis(4-chlorobenzoyl)-2,7-dimethoxynaphthalene (Nakaema *et al.*, 2007), followed by conversion of hydroxy groups to benzyloxy ones. The molecular structure of the title compound is illustrated in Fig. 1. The two intervenient benzene rings, A (C12—C17) and B (C19—C24), are in a *syn* orientation with respect to the naphthalene ring system (C1—C10), and make the dihedral angles of 57.12 (6) and 85.74 (6)°, respectively, with the naphthalene ring system. Furthermore, the dihedral angles of the two benzene rings in the benzyloxy groups, C (C28—C33) and D (C35—C40), against the naphthalene ring system are 75.34 (6) and 83.95 (7)°, respectively. Besides, the interplanar angle between benzene rings A (C12—C17) and B (C19—C24) is smaller than that between benzene ring C (C28—C33) and D (C35—C40) [31.39 (7) and 84.68 (9)°, respectively].

In the molecular packing, the C—H···Cl interactions between the hydrogen atoms of the methylene moiety and the chloro atoms of the 4-chlorophenyl rings of the adjacent molecules are observed atom along the *a* axis [C27—H27A···Cl1^i^ = 2.66 Å](Fig. 2). Furthermore, C—H···π interactions between the hydrogen atom of the benzene ring A and the π-system of the benzene ring D (with centroid *Cg*6) is also observed (C16—H16···*Cg*6^ii^ = 2.70 Å; Table 1), resulting in the formation of dimeric units having crystallographic inversion centre (Fig. 3).

### Experimental

To a solution of the pinacol compound, 1,2-bis(4-chlorophenyl)-1,2-dihydroxy-3,8-dimethoxyacenaphthene (0.1 mmol, 46 mg) in DMAc (0.1 ml), a mixture of benzyl bromide (0.22 mmol, 34 mg), NaH (0.22 mmol, 48 mg), and tetrabutylammonium iodide (0.01 mmol, 2 mg) was added by portions at r.t. After the reaction mixture was stirred for 3 h, it was poured into ice-cold water (10 ml). The aqueous layer was extracted with CHCl_3_ (10 ml ×3). The combined extracts were washed with 2 *M* aqueous HCl followed by washing with brine. The organic layers thus obtained were dried over anhydrous MgSO_4_. The solvent was removed under reduced pressure to give cake (yield 27 mg, 42%). The crude material was purified by recrystallization from CHCl_3_/ethanol to give the title compound as colorless platelets (isolated yield, 38%). Spectroscopic Data: ^1^H NMR (300 MHz, CDCl_3_)δ;7.88, (d, 2H), 7.19–7.26(m, 12H), 6.84 (d, 8H), 4.74–4.84(m, 4H), 3.68(s, 6H); ^13^C NMR(75 MHz, CDCl_3_); 154.5, 142.0, 140.3, 140.0, 132.1, 128.9, 128.9, 127.9, 127.3, 126.8, 126.7, 122.4, 122.3, 113.6, 96.7, 69.1, 55,7; IR (KBr);1623, 1502, 1259 cm^-1^; Anal. Calcd for C_40_H_32_Cl_2_O_4_; C, 74.19; H, 4.98. Found: C, 74.176; H, 5.160%; m.p.=203.0–204.0 K.

### Refinement

All H atoms were found in a difference map and were subsequently refined as riding atoms, with C—H = 0.95 (aromatic) and 0.98 (methyl) Å, and with *U*_ĩso_(H) = 1.2 *U*_eq_(C).

### Crystal data

**Table:**

C_40_H_32_Cl_2_O_4_	*Z* = 2
*M**_r_* = 647.56	*F*(000) = 676
Triclinic, *P*1	*D*_x_ = 1.332 Mg m^−^^3^
Hall symbol: -P 1	Cu *K*α radiation, λ = 1.54187 Å
*a* = 10.9773 (2) Å	Cell parameters from 29110 reflections
*b* = 12.6514 (2) Å	θ = 3.7–68.2°
*c* = 12.9171 (2) Å	µ = 2.15 mm^−^^1^
α = 102.387 (1)°	*T* = 193 K
β = 104.899 (1)°	Block, colorless
γ = 103.306 (1)°	0.50 × 0.30 × 0.20 mm
*V* = 1614.04 (5) Å^3^	

### Data collection

**Table:**

Rigaku R-AXIS RAPID diffractometer	5828 independent reflections
Radiation source: rotating anode	5503 reflections with *I* > 2σ(*I*)
graphite	*R*_int_ = 0.061
Detector resolution: 10.000 pixels mm^-1^	θ_max_ = 68.2°, θ_min_ = 3.7°
ω scans	*h* = −13→12
Absorption correction: numerical (*NUMABS*; Higashi,1999)	*k* = −15→15
*T*_min_ = 0.414, *T*_max_ = 0.674	*l* = −15→15
30622 measured reflections	

### Refinement

**Table:**

Refinement on *F*^2^	Secondary atom site location: difference Fourier map
Least-squares matrix: full	Hydrogen site location: inferred from neighbouring sites
*R*[*F*^2^ > 2σ(*F*^2^)] = 0.037	H-atom parameters constrained
*wR*(*F*^2^) = 0.107	*w* = 1/[σ^2^(*F*_o_^2^) + (0.0623*P*)^2^ + 0.4586*P*] where *P* = (*F*_o_^2^ + 2*F*_c_^2^)/3
*S* = 1.06	(Δ/σ)_max_ = 0.002
5828 reflections	Δρ_max_ = 0.43 e Å^−^^3^
418 parameters	Δρ_min_ = −0.31 e Å^−^^3^
0 restraints	Extinction correction: *SHELXL97* (Sheldrick, 2008), Fc^*^=kFc[1+0.001xFc^2^λ^3^/sin(2θ)]^-1/4^
Primary atom site location: structure-invariant direct methods	Extinction coefficient: 0.0082 (4)

### Special details

**Table:**

Geometry. All e.s.d.'s (except the e.s.d. in the dihedral angle between two l.s. planes) are estimated using the full covariance matrix. The cell e.s.d.'s are taken into account individually in the estimation of e.s.d.'s in distances, angles and torsion angles; correlations between e.s.d.'s in cell parameters are only used when they are defined by crystal symmetry. An approximate (isotropic) treatment of cell e.s.d.'s is used for estimating e.s.d.'s involving l.s. planes.
Refinement. Refinement of *F*^2^ against ALL reflections. The weighted *R*-factor *wR* and goodness of fit *S* are based on *F*^2^, conventional *R*-factors *R* are based on *F*, with *F* set to zero for negative *F*^2^. The threshold expression of *F*^2^ > σ(*F*^2^) is used only for calculating *R*-factors(gt) *etc*. and is not relevant to the choice of reflections for refinement. *R*-factors based on *F*^2^ are statistically about twice as large as those based on *F*, and *R*- factors based on ALL data will be even larger.

### Fractional atomic coordinates and isotropic or equivalent isotropic displacement parameters (Å^2^)

**Table:**

	*x*	*y*	*z*	*U*_iso_*/*U*_eq_	
Cl1	1.11323 (4)	0.16739 (4)	0.15815 (4)	0.05437 (15)	
Cl2	1.10086 (4)	0.63681 (3)	0.37022 (3)	0.04790 (14)	
O1	0.49403 (9)	0.07269 (7)	0.17290 (8)	0.0276 (2)	
O2	0.51556 (9)	0.25848 (8)	0.10705 (7)	0.0285 (2)	
O3	0.70682 (12)	0.05722 (9)	0.43438 (9)	0.0405 (3)	
O4	0.39937 (12)	0.43218 (10)	0.22526 (10)	0.0437 (3)	
C1	0.59210 (13)	0.17780 (11)	0.36826 (11)	0.0283 (3)	
C2	0.63214 (15)	0.12827 (12)	0.45074 (12)	0.0328 (3)	
C3	0.59278 (17)	0.15073 (14)	0.54799 (12)	0.0397 (4)	
H3	0.6227	0.1183	0.6059	0.048*	
C4	0.51272 (17)	0.21801 (14)	0.55995 (13)	0.0413 (4)	
H4	0.4864	0.2298	0.6250	0.050*	
C5	0.46860 (15)	0.27021 (12)	0.47695 (12)	0.0354 (3)	
C6	0.38935 (16)	0.34403 (13)	0.47836 (14)	0.0409 (4)	
H6	0.3545	0.3588	0.5382	0.049*	
C7	0.36209 (16)	0.39443 (13)	0.39523 (14)	0.0403 (4)	
H7	0.3070	0.4423	0.3977	0.048*	
C8	0.41422 (14)	0.37692 (12)	0.30514 (13)	0.0337 (3)	
C9	0.48695 (13)	0.30165 (11)	0.29829 (11)	0.0284 (3)	
C10	0.51254 (13)	0.24915 (11)	0.38316 (11)	0.0293 (3)	
C11	0.60405 (13)	0.16494 (11)	0.25134 (10)	0.0256 (3)	
C12	0.85295 (14)	0.19940 (12)	0.31986 (12)	0.0317 (3)	
H12	0.8545	0.2269	0.3948	0.038*	
C13	0.73237 (13)	0.15671 (11)	0.23323 (11)	0.0262 (3)	
C14	0.97044 (14)	0.20211 (13)	0.29780 (13)	0.0367 (3)	
H14	1.0524	0.2308	0.3570	0.044*	
C15	0.96649 (14)	0.16232 (12)	0.18812 (13)	0.0347 (3)	
C16	0.84865 (15)	0.11863 (12)	0.10077 (12)	0.0330 (3)	
H16	0.8476	0.0912	0.0260	0.040*	
C17	0.73205 (14)	0.11564 (12)	0.12420 (11)	0.0295 (3)	
H17	0.6503	0.0850	0.0648	0.035*	
C18	0.57022 (13)	0.27657 (11)	0.22371 (11)	0.0263 (3)	
C19	0.69953 (13)	0.37403 (11)	0.26034 (11)	0.0270 (3)	
C20	0.75744 (14)	0.44304 (11)	0.37078 (11)	0.0295 (3)	
H20	0.7123	0.4341	0.4235	0.035*	
C21	0.87998 (15)	0.52457 (12)	0.40500 (12)	0.0329 (3)	
H21	0.9185	0.5711	0.4804	0.039*	
C22	0.94520 (14)	0.53719 (12)	0.32793 (12)	0.0337 (3)	
C23	0.88904 (15)	0.47151 (13)	0.21747 (12)	0.0358 (3)	
H23	0.9340	0.4816	0.1649	0.043*	
C24	0.76659 (14)	0.39090 (12)	0.18422 (12)	0.0319 (3)	
H24	0.7275	0.3462	0.1081	0.038*	
C25	0.77576 (19)	0.02741 (16)	0.52774 (14)	0.0494 (4)	
H25A	0.8308	0.0966	0.5881	0.059*	
H25B	0.8320	−0.0168	0.5052	0.059*	
H25C	0.7119	−0.0181	0.5541	0.059*	
C26	0.3045 (2)	0.49310 (18)	0.2163 (2)	0.0608 (5)	
H26A	0.2179	0.4423	0.2075	0.073*	
H26B	0.2984	0.5218	0.1511	0.073*	
H26C	0.3319	0.5570	0.2842	0.073*	
C27	0.49115 (14)	−0.03802 (11)	0.18391 (12)	0.0310 (3)	
H27A	0.5499	−0.0313	0.2589	0.037*	
H27B	0.5234	−0.0781	0.1270	0.037*	
C28	0.35182 (14)	−0.10438 (11)	0.16839 (11)	0.0281 (3)	
C29	0.26258 (15)	−0.05050 (13)	0.19605 (12)	0.0330 (3)	
H29	0.2898	0.0298	0.2259	0.040*	
C30	0.13410 (16)	−0.11310 (15)	0.18040 (13)	0.0405 (4)	
H30	0.0738	−0.0754	0.1991	0.049*	
C31	0.09343 (17)	−0.23016 (15)	0.13764 (15)	0.0476 (4)	
H31	0.0054	−0.2729	0.1265	0.057*	
C32	0.18202 (18)	−0.28417 (14)	0.11136 (15)	0.0476 (4)	
H32	0.1549	−0.3646	0.0830	0.057*	
C33	0.31026 (16)	−0.22221 (12)	0.12593 (13)	0.0366 (3)	
H33	0.3700	−0.2604	0.1068	0.044*	
C34	0.37626 (13)	0.20801 (12)	0.05308 (12)	0.0322 (3)	
H34A	0.3278	0.2371	0.1019	0.039*	
H34B	0.3530	0.1246	0.0382	0.039*	
C35	0.33847 (13)	0.23730 (12)	−0.05534 (12)	0.0290 (3)	
C36	0.23404 (15)	0.16125 (14)	−0.14550 (13)	0.0390 (3)	
H36	0.1883	0.0911	−0.1383	0.047*	
C37	0.19564 (18)	0.18624 (17)	−0.24597 (15)	0.0508 (4)	
H37	0.1233	0.1337	−0.3068	0.061*	
C38	0.26201 (19)	0.28700 (18)	−0.25794 (15)	0.0533 (5)	
H38	0.2359	0.3039	−0.3270	0.064*	
C39	0.3665 (2)	0.36332 (17)	−0.16918 (18)	0.0548 (5)	
H39	0.4127	0.4328	−0.1772	0.066*	
C40	0.40430 (17)	0.33878 (14)	−0.06819 (15)	0.0421 (4)	
H40	0.4760	0.3920	−0.0072	0.051*	

### Atomic displacement parameters (Å^2^)

**Table:**

	*U*^11^	*U*^22^	*U*^33^	*U*^12^	*U*^13^	*U*^23^
Cl1	0.0299 (2)	0.0611 (3)	0.0746 (3)	0.01170 (18)	0.0276 (2)	0.0141 (2)
Cl2	0.0353 (2)	0.0478 (2)	0.0438 (2)	−0.01135 (17)	0.00949 (17)	0.00916 (17)
O1	0.0228 (5)	0.0269 (5)	0.0287 (5)	0.0040 (4)	0.0044 (4)	0.0078 (4)
O2	0.0223 (5)	0.0362 (5)	0.0248 (5)	0.0045 (4)	0.0066 (4)	0.0106 (4)
O3	0.0484 (7)	0.0467 (6)	0.0331 (5)	0.0200 (5)	0.0126 (5)	0.0194 (5)
O4	0.0443 (6)	0.0435 (6)	0.0565 (7)	0.0224 (5)	0.0227 (5)	0.0232 (5)
C1	0.0249 (7)	0.0301 (6)	0.0269 (7)	0.0019 (5)	0.0100 (5)	0.0072 (5)
C2	0.0330 (7)	0.0331 (7)	0.0287 (7)	0.0028 (6)	0.0095 (6)	0.0100 (6)
C3	0.0453 (9)	0.0429 (8)	0.0285 (7)	0.0043 (7)	0.0130 (6)	0.0140 (6)
C4	0.0463 (9)	0.0443 (8)	0.0305 (7)	0.0018 (7)	0.0203 (7)	0.0080 (6)
C5	0.0343 (8)	0.0344 (7)	0.0330 (7)	0.0001 (6)	0.0172 (6)	0.0042 (6)
C6	0.0388 (8)	0.0399 (8)	0.0418 (8)	0.0051 (7)	0.0234 (7)	0.0021 (7)
C7	0.0337 (8)	0.0353 (7)	0.0509 (9)	0.0093 (6)	0.0205 (7)	0.0031 (7)
C8	0.0285 (7)	0.0303 (7)	0.0397 (8)	0.0047 (6)	0.0127 (6)	0.0072 (6)
C9	0.0233 (6)	0.0287 (6)	0.0296 (7)	0.0028 (5)	0.0099 (5)	0.0052 (5)
C10	0.0252 (7)	0.0285 (6)	0.0289 (7)	0.0000 (5)	0.0103 (5)	0.0045 (5)
C11	0.0230 (6)	0.0280 (6)	0.0234 (6)	0.0041 (5)	0.0068 (5)	0.0077 (5)
C12	0.0279 (7)	0.0360 (7)	0.0269 (7)	0.0068 (6)	0.0054 (6)	0.0078 (6)
C13	0.0242 (7)	0.0271 (6)	0.0264 (6)	0.0055 (5)	0.0075 (5)	0.0094 (5)
C14	0.0232 (7)	0.0399 (8)	0.0394 (8)	0.0052 (6)	0.0031 (6)	0.0094 (6)
C15	0.0254 (7)	0.0350 (7)	0.0475 (8)	0.0089 (6)	0.0166 (6)	0.0139 (6)
C16	0.0321 (7)	0.0364 (7)	0.0329 (7)	0.0090 (6)	0.0152 (6)	0.0106 (6)
C17	0.0257 (7)	0.0346 (7)	0.0262 (6)	0.0061 (5)	0.0069 (5)	0.0095 (5)
C18	0.0237 (6)	0.0293 (6)	0.0255 (6)	0.0062 (5)	0.0083 (5)	0.0087 (5)
C19	0.0247 (7)	0.0280 (6)	0.0286 (6)	0.0065 (5)	0.0085 (5)	0.0104 (5)
C20	0.0297 (7)	0.0309 (7)	0.0284 (7)	0.0071 (6)	0.0114 (6)	0.0090 (5)
C21	0.0326 (7)	0.0310 (7)	0.0295 (7)	0.0046 (6)	0.0068 (6)	0.0067 (6)
C22	0.0273 (7)	0.0315 (7)	0.0361 (7)	0.0006 (6)	0.0068 (6)	0.0109 (6)
C23	0.0326 (8)	0.0403 (8)	0.0335 (7)	0.0031 (6)	0.0140 (6)	0.0135 (6)
C24	0.0305 (7)	0.0349 (7)	0.0273 (7)	0.0039 (6)	0.0093 (6)	0.0092 (6)
C25	0.0537 (10)	0.0538 (10)	0.0401 (9)	0.0176 (8)	0.0058 (8)	0.0224 (8)
C26	0.0599 (12)	0.0563 (11)	0.0831 (14)	0.0353 (10)	0.0266 (11)	0.0307 (10)
C27	0.0268 (7)	0.0288 (7)	0.0348 (7)	0.0087 (5)	0.0066 (6)	0.0079 (6)
C28	0.0288 (7)	0.0323 (7)	0.0225 (6)	0.0071 (6)	0.0063 (5)	0.0115 (5)
C29	0.0328 (8)	0.0370 (7)	0.0301 (7)	0.0097 (6)	0.0115 (6)	0.0107 (6)
C30	0.0335 (8)	0.0534 (9)	0.0399 (8)	0.0117 (7)	0.0176 (7)	0.0189 (7)
C31	0.0348 (8)	0.0531 (10)	0.0529 (10)	−0.0003 (7)	0.0148 (7)	0.0258 (8)
C32	0.0456 (10)	0.0338 (8)	0.0556 (10)	−0.0004 (7)	0.0111 (8)	0.0171 (7)
C33	0.0385 (8)	0.0324 (7)	0.0375 (8)	0.0090 (6)	0.0094 (6)	0.0122 (6)
C34	0.0227 (7)	0.0374 (7)	0.0328 (7)	0.0023 (6)	0.0065 (6)	0.0130 (6)
C35	0.0245 (7)	0.0326 (7)	0.0339 (7)	0.0117 (5)	0.0110 (6)	0.0123 (6)
C36	0.0315 (8)	0.0426 (8)	0.0396 (8)	0.0075 (6)	0.0057 (6)	0.0159 (7)
C37	0.0410 (9)	0.0684 (11)	0.0380 (9)	0.0157 (8)	0.0026 (7)	0.0182 (8)
C38	0.0527 (11)	0.0813 (13)	0.0447 (9)	0.0325 (10)	0.0188 (8)	0.0392 (9)
C39	0.0545 (11)	0.0576 (11)	0.0680 (12)	0.0182 (9)	0.0243 (9)	0.0425 (10)
C40	0.0414 (9)	0.0371 (8)	0.0464 (9)	0.0072 (7)	0.0110 (7)	0.0183 (7)

### Geometric parameters (Å, °)

**Table:**

Cl1—C15	1.7414 (15)	C20—C21	1.389 (2)
Cl2—C22	1.7435 (14)	C20—H20	0.9500
O1—C27	1.4320 (16)	C21—C22	1.382 (2)
O1—C11	1.4359 (15)	C21—H21	0.9500
O2—C18	1.4167 (15)	C22—C23	1.382 (2)
O2—C34	1.4295 (16)	C23—C24	1.384 (2)
O3—C2	1.3680 (19)	C23—H23	0.9500
O3—C25	1.4235 (18)	C24—H24	0.9500
O4—C8	1.3621 (19)	C25—H25A	0.9800
O4—C26	1.428 (2)	C25—H25B	0.9800
C1—C2	1.376 (2)	C25—H25C	0.9800
C1—C10	1.410 (2)	C26—H26A	0.9800
C1—C11	1.5267 (18)	C26—H26B	0.9800
C2—C3	1.424 (2)	C26—H26C	0.9800
C3—C4	1.371 (2)	C27—C28	1.5044 (19)
C3—H3	0.9500	C27—H27A	0.9900
C4—C5	1.416 (2)	C27—H27B	0.9900
C4—H4	0.9500	C28—C29	1.391 (2)
C5—C10	1.412 (2)	C28—C33	1.392 (2)
C5—C6	1.416 (2)	C29—C30	1.388 (2)
C6—C7	1.366 (2)	C29—H29	0.9500
C6—H6	0.9500	C30—C31	1.384 (2)
C7—C8	1.422 (2)	C30—H30	0.9500
C7—H7	0.9500	C31—C32	1.380 (3)
C8—C9	1.379 (2)	C31—H31	0.9500
C9—C10	1.4006 (19)	C32—C33	1.387 (2)
C9—C18	1.5230 (19)	C32—H32	0.9500
C11—C13	1.5089 (18)	C33—H33	0.9500
C11—C18	1.6277 (18)	C34—C35	1.5050 (19)
C12—C14	1.385 (2)	C34—H34A	0.9900
C12—C13	1.3949 (18)	C34—H34B	0.9900
C12—H12	0.9500	C35—C36	1.386 (2)
C13—C17	1.3913 (19)	C35—C40	1.387 (2)
C14—C15	1.384 (2)	C36—C37	1.385 (2)
C14—H14	0.9500	C36—H36	0.9500
C15—C16	1.380 (2)	C37—C38	1.376 (3)
C16—C17	1.383 (2)	C37—H37	0.9500
C16—H16	0.9500	C38—C39	1.379 (3)
C17—H17	0.9500	C38—H38	0.9500
C18—C19	1.5355 (18)	C39—C40	1.387 (2)
C19—C24	1.394 (2)	C39—H39	0.9500
C19—C20	1.3947 (19)	C40—H40	0.9500
			
C27—O1—C11	115.90 (10)	C22—C21—H21	120.4
C18—O2—C34	119.75 (10)	C20—C21—H21	120.4
C2—O3—C25	118.69 (13)	C23—C22—C21	120.94 (13)
C8—O4—C26	118.61 (14)	C23—C22—Cl2	119.27 (12)
C2—C1—C10	118.46 (13)	C21—C22—Cl2	119.79 (11)
C2—C1—C11	133.47 (13)	C22—C23—C24	119.35 (14)
C10—C1—C11	107.80 (11)	C22—C23—H23	120.3
O3—C2—C1	118.01 (13)	C24—C23—H23	120.3
O3—C2—C3	122.65 (13)	C23—C24—C19	121.21 (13)
C1—C2—C3	119.32 (14)	C23—C24—H24	119.4
C4—C3—C2	121.51 (14)	C19—C24—H24	119.4
C4—C3—H3	119.2	O3—C25—H25A	109.5
C2—C3—H3	119.2	O3—C25—H25B	109.5
C3—C4—C5	121.07 (14)	H25A—C25—H25B	109.5
C3—C4—H4	119.5	O3—C25—H25C	109.5
C5—C4—H4	119.5	H25A—C25—H25C	109.5
C10—C5—C4	116.08 (14)	H25B—C25—H25C	109.5
C10—C5—C6	116.03 (14)	O4—C26—H26A	109.5
C4—C5—C6	127.84 (14)	O4—C26—H26B	109.5
C7—C6—C5	121.22 (14)	H26A—C26—H26B	109.5
C7—C6—H6	119.4	O4—C26—H26C	109.5
C5—C6—H6	119.4	H26A—C26—H26C	109.5
C6—C7—C8	121.50 (14)	H26B—C26—H26C	109.5
C6—C7—H7	119.3	O1—C27—C28	109.42 (11)
C8—C7—H7	119.3	O1—C27—H27A	109.8
O4—C8—C9	117.19 (13)	C28—C27—H27A	109.8
O4—C8—C7	123.92 (14)	O1—C27—H27B	109.8
C9—C8—C7	118.88 (14)	C28—C27—H27B	109.8
C8—C9—C10	118.99 (13)	H27A—C27—H27B	108.2
C8—C9—C18	131.49 (13)	C29—C28—C33	118.75 (14)
C10—C9—C18	108.56 (12)	C29—C28—C27	121.35 (12)
C9—C10—C1	113.21 (12)	C33—C28—C27	119.90 (13)
C9—C10—C5	123.21 (14)	C30—C29—C28	120.52 (14)
C1—C10—C5	123.50 (13)	C30—C29—H29	119.7
O1—C11—C13	111.10 (10)	C28—C29—H29	119.7
O1—C11—C1	108.72 (10)	C31—C30—C29	120.34 (16)
C13—C11—C1	119.20 (11)	C31—C30—H30	119.8
O1—C11—C18	103.24 (9)	C29—C30—H30	119.8
C13—C11—C18	111.09 (10)	C32—C31—C30	119.38 (15)
C1—C11—C18	102.01 (10)	C32—C31—H31	120.3
C14—C12—C13	120.71 (13)	C30—C31—H31	120.3
C14—C12—H12	119.6	C31—C32—C33	120.65 (15)
C13—C12—H12	119.6	C31—C32—H32	119.7
C17—C13—C12	118.78 (13)	C33—C32—H32	119.7
C17—C13—C11	118.42 (11)	C32—C33—C28	120.34 (15)
C12—C13—C11	122.46 (12)	C32—C33—H33	119.8
C15—C14—C12	118.91 (13)	C28—C33—H33	119.8
C15—C14—H14	120.5	O2—C34—C35	108.48 (11)
C12—C14—H14	120.5	O2—C34—H34A	110.0
C16—C15—C14	121.71 (13)	C35—C34—H34A	110.0
C16—C15—Cl1	118.59 (12)	O2—C34—H34B	110.0
C14—C15—Cl1	119.70 (12)	C35—C34—H34B	110.0
C15—C16—C17	118.71 (13)	H34A—C34—H34B	108.4
C15—C16—H16	120.6	C36—C35—C40	118.50 (14)
C17—C16—H16	120.6	C36—C35—C34	119.35 (13)
C16—C17—C13	121.17 (13)	C40—C35—C34	122.14 (13)
C16—C17—H17	119.4	C37—C36—C35	120.80 (15)
C13—C17—H17	119.4	C37—C36—H36	119.6
O2—C18—C9	118.69 (11)	C35—C36—H36	119.6
O2—C18—C19	104.84 (10)	C38—C37—C36	120.20 (17)
C9—C18—C19	110.59 (11)	C38—C37—H37	119.9
O2—C18—C11	111.71 (10)	C36—C37—H37	119.9
C9—C18—C11	101.83 (10)	C37—C38—C39	119.70 (15)
C19—C18—C11	109.05 (10)	C37—C38—H38	120.2
C24—C19—C20	118.16 (13)	C39—C38—H38	120.2
C24—C19—C18	120.05 (12)	C38—C39—C40	120.14 (16)
C20—C19—C18	121.67 (12)	C38—C39—H39	119.9
C21—C20—C19	121.12 (13)	C40—C39—H39	119.9
C21—C20—H20	119.4	C35—C40—C39	120.66 (16)
C19—C20—H20	119.4	C35—C40—H40	119.7
C22—C21—C20	119.19 (13)	C39—C40—H40	119.7
			
C25—O3—C2—C1	164.83 (14)	C11—C13—C17—C16	172.21 (12)
C25—O3—C2—C3	−16.4 (2)	C34—O2—C18—C9	−31.50 (16)
C10—C1—C2—O3	178.58 (12)	C34—O2—C18—C19	−155.53 (11)
C11—C1—C2—O3	5.4 (2)	C34—O2—C18—C11	86.51 (14)
C10—C1—C2—C3	−0.2 (2)	C8—C9—C18—O2	−48.2 (2)
C11—C1—C2—C3	−173.39 (14)	C10—C9—C18—O2	143.42 (12)
O3—C2—C3—C4	−176.86 (14)	C8—C9—C18—C19	72.93 (18)
C1—C2—C3—C4	1.9 (2)	C10—C9—C18—C19	−95.42 (12)
C2—C3—C4—C5	−1.6 (2)	C8—C9—C18—C11	−171.29 (14)
C3—C4—C5—C10	−0.4 (2)	C10—C9—C18—C11	20.36 (13)
C3—C4—C5—C6	−178.05 (15)	O1—C11—C18—O2	−39.72 (13)
C10—C5—C6—C7	−2.3 (2)	C13—C11—C18—O2	79.42 (13)
C4—C5—C6—C7	175.37 (15)	C1—C11—C18—O2	−152.50 (10)
C5—C6—C7—C8	−1.3 (2)	O1—C11—C18—C9	87.97 (11)
C26—O4—C8—C9	167.89 (15)	C13—C11—C18—C9	−152.88 (10)
C26—O4—C8—C7	−13.6 (2)	C1—C11—C18—C9	−24.81 (12)
C6—C7—C8—O4	−174.33 (14)	O1—C11—C18—C19	−155.13 (10)
C6—C7—C8—C9	4.1 (2)	C13—C11—C18—C19	−35.98 (14)
O4—C8—C9—C10	175.50 (12)	C1—C11—C18—C19	92.09 (12)
C7—C8—C9—C10	−3.1 (2)	O2—C18—C19—C24	−27.44 (16)
O4—C8—C9—C18	8.1 (2)	C9—C18—C19—C24	−156.49 (12)
C7—C8—C9—C18	−170.44 (14)	C11—C18—C19—C24	92.32 (14)
C8—C9—C10—C1	−177.65 (12)	O2—C18—C19—C20	156.65 (12)
C18—C9—C10—C1	−7.61 (15)	C9—C18—C19—C20	27.60 (17)
C8—C9—C10—C5	−0.7 (2)	C11—C18—C19—C20	−83.59 (15)
C18—C9—C10—C5	169.36 (12)	C24—C19—C20—C21	−1.5 (2)
C2—C1—C10—C9	175.18 (12)	C18—C19—C20—C21	174.45 (12)
C11—C1—C10—C9	−10.03 (15)	C19—C20—C21—C22	0.0 (2)
C2—C1—C10—C5	−1.8 (2)	C20—C21—C22—C23	1.3 (2)
C11—C1—C10—C5	173.01 (12)	C20—C21—C22—Cl2	−178.48 (11)
C4—C5—C10—C9	−174.59 (13)	C21—C22—C23—C24	−1.0 (2)
C6—C5—C10—C9	3.4 (2)	Cl2—C22—C23—C24	178.78 (12)
C4—C5—C10—C1	2.1 (2)	C22—C23—C24—C19	−0.6 (2)
C6—C5—C10—C1	−179.96 (13)	C20—C19—C24—C23	1.8 (2)
C27—O1—C11—C13	65.90 (14)	C18—C19—C24—C23	−174.20 (13)
C27—O1—C11—C1	−67.17 (14)	C11—O1—C27—C28	138.79 (11)
C27—O1—C11—C18	−174.96 (10)	O1—C27—C28—C29	−29.00 (17)
C2—C1—C11—O1	86.75 (17)	O1—C27—C28—C33	151.23 (12)
C10—C1—C11—O1	−86.93 (12)	C33—C28—C29—C30	−0.7 (2)
C2—C1—C11—C13	−41.9 (2)	C27—C28—C29—C30	179.54 (13)
C10—C1—C11—C13	144.40 (12)	C28—C29—C30—C31	0.4 (2)
C2—C1—C11—C18	−164.62 (15)	C29—C30—C31—C32	0.4 (2)
C10—C1—C11—C18	21.69 (13)	C30—C31—C32—C33	−0.9 (3)
C14—C12—C13—C17	0.7 (2)	C31—C32—C33—C28	0.6 (2)
C14—C12—C13—C11	−172.46 (13)	C29—C28—C33—C32	0.2 (2)
O1—C11—C13—C17	35.72 (15)	C27—C28—C33—C32	179.96 (14)
C1—C11—C13—C17	163.29 (12)	C18—O2—C34—C35	159.31 (11)
C18—C11—C13—C17	−78.61 (14)	O2—C34—C35—C36	148.56 (13)
O1—C11—C13—C12	−151.08 (12)	O2—C34—C35—C40	−31.88 (19)
C1—C11—C13—C12	−23.51 (18)	C40—C35—C36—C37	−0.4 (2)
C18—C11—C13—C12	94.59 (14)	C34—C35—C36—C37	179.15 (15)
C13—C12—C14—C15	0.4 (2)	C35—C36—C37—C38	0.7 (3)
C12—C14—C15—C16	−1.0 (2)	C36—C37—C38—C39	−0.4 (3)
C12—C14—C15—Cl1	178.80 (11)	C37—C38—C39—C40	−0.2 (3)
C14—C15—C16—C17	0.5 (2)	C36—C35—C40—C39	−0.1 (2)
Cl1—C15—C16—C17	−179.31 (11)	C34—C35—C40—C39	−179.71 (16)
C15—C16—C17—C13	0.7 (2)	C38—C39—C40—C35	0.5 (3)
C12—C13—C17—C16	−1.2 (2)		

### Hydrogen-bond geometry (Å, °)

**Table:**

Cg6 is the centroid of the C35–C40 ring.

**Table:**

*D*—H···*A*	*D*—H	H···*A*	*D*···*A*	*D*—H···*A*
C34—H34A···Cl1^i^	0.99	2.66	3.4748 (16)	140
C16—H16···Cg6^ii^	0.95	2.70	3.3962 (16)	131

Symmetry codes: (i) *x*−1, *y*, *z*; (ii) −*x*+1, −*y*, −*z*.

## References

[bb1] Burla, M. C., Caliandro, R., Camalli, M., Carrozzini, B., Cascarano, G. L., De Caro, L., Giacovazzo, C., Polidori, G. & Spagna, R. (2005). *J. Appl. Cryst.* **38**, 381–388.

[bb2] Burnett, M. N. & Johnson, C. K. (1996). *ORTEPIII* Report ORNL-6865. Oak Ridge National Laboratory. Tennessee, USA.

[bb3] Higashi, T. (1999). *NUMABS* Rigaku Corporation, Tokyo, Japan.

[bb4] Hijikata, D., Takada, T., Nagasawa, A., Okamoto, A. & Yonezawa, N. (2010). *Acta Cryst.* E**66**, o2902–o2903. 10.1107/S1600536810042170 PMC3009209 21589079

[bb5] Mitsui, R., Nagasawa, A., Noguchi, K., Okamoto, A. & Yonezawa, N. (2010). *Acta Cryst.* E**66**, o1790. 10.1107/S1600536810024074 PMC3007080 21588000

[bb6] Nakaema, K., Okamoto, A., Noguchi, K. & Yonezawa, N. (2007). *Acta Cryst.* E**63**, o4120.

[bb7] Okamoto, A. & Yonezawa, N. (2009). *Chem* *Lett* **38**, 914–915

[bb8] Rigaku (1998). *PROCESS-AUTO* Rigaku Corporation, Tokyo, Japan.

[bb9] Rigaku/MSC (2004). *CrystalStructure* Rigaku/MSC, The Woodlands, Texas, USA.

[bb10] Sheldrick, G. M. (2008). *Acta Cryst.* A**64**, 112–122. 10.1107/S0108767307043930 18156677

[bb11] Watanabe, S., Nagasawa, A., Okamoto, A., Noguchi, K. & Yonezawa, N. (2010*a*). *Acta Cryst.* E**66**, o329. 10.1107/S1600536810000486 PMC2979763 21579759

[bb12] Watanabe, S., Nakaema, K., Muto, T., Okamoto, A. & Yonezawa, N. (2010*b*). *Acta Cryst.* E**66**, o403. 10.1107/S1600536810001819 PMC2979875 21579823

